# The S Protein of Group B *Streptococcus* Is a Critical Virulence Determinant That Impacts the Cell Surface Virulome

**DOI:** 10.3389/fmicb.2021.729308

**Published:** 2021-10-14

**Authors:** Anaamika Campeau, Satoshi Uchiyama, Concepcion Sanchez, Consuelo Sauceda, Victor Nizet, David J. Gonzalez

**Affiliations:** ^1^Department of Pharmacology, University of California San Diego, La Jolla, CA, United States; ^2^Skaggs School of Pharmacy and Pharmaceutical Sciences, University of California San Diego, La Jolla, CA, United States; ^3^Center for Microbiome Innovation, University of California San Diego, La Jolla, CA, United States; ^4^Department of Pediatrics, University of California San Diego, La Jolla, CA, United States

**Keywords:** group B *Streptococcus*, S protein, surfome, mass spectrometry, capsule

## Abstract

Group B *Streptococcus* (GBS, *S. agalactiae*) is a human commensal and occasional pathogen that remains a leading cause of neonatal sepsis and meningitis with increasing disease burden in adult populations. Although programs for universal screening in pregnancy to guide intrapartum prophylaxis have reduced GBS invasive disease burden resulting from mother-to-newborn transfer during birth, better knowledge of disease mechanisms may elucidate new strategies to reduce antibiotic exposure. In our efforts to expand the knowledge base required for targeted anti-virulence therapies, we identified a GBS homolog for a recently identified virulence determinant of group A *Streptococcus*, S protein, and evaluated its role in GBS pathogenesis. A GBS S protein deletion mutant, Δ*ess*, showed altered cell-surface properties compared to the WT parent strain, including defective retention of its surface polysaccharide. Quantitative proteome analysis of enzymatically shaved surface epitopes of the GBS Δ*ess* mutant revealed a dysregulated cell surface virulome, with reduced abundance of several protein and glycoprotein components. The Δ*ess* mutant showed markedly attenuated virulence in a murine model of GBS systemic infection, with increased proteasome activity detected in the spleens of animals infected with the Δ*ess* mutant. These results expand the key roles S protein plays in streptococcal pathogenesis and introduces a new GBS virulence determinant and potential target for therapy development.

## Introduction

Group B *Streptococcus* (GBS), or *Streptococcus agalactiae*, remains a major cause of neonatal morbidity and mortality across the globe. A commensal in an estimated 25% of the healthy adult population, GBS can be transmitted from mother to child during delivery (Stoll et al., 2011; Seale et al., 2017). Universal maternal screening protocols and intrapartum antibiotic prophylaxis have made GBS-induced preterm birth and early onset GBS infection (i.e., infections that occur during the first week of life) less common (Renner et al., 2006; Seedat et al., 2019). However, GBS infections are increasingly reported in scenarios where maternal prophylaxis is ineffective, such as in infants beyond the first week of life, pregnant women, and older or immunocompromised adults (Farley, 2001). Additionally, the use of intrapartum antibiotics is increasingly scrutinized given the selective pressure for antibiotic resistance and deleterious effects of antibiotic administration on maternal-to-neonatal microbiome transfer, which is recognized for its importance in early neonatal health and immunity (Cassidy-Bushrow et al., 2016; Kolter and Henneke, 2017; Stearns et al., 2017; Tapiainen et al., 2019).

One potential alternative approach to intrapartum use of antibiotics in GBS-colonized mothers is the use of pharmacological agents that target virulence factors. The development of anti-virulence strategies requires a comprehensive understanding of the molecular mechanisms by which a pathogen causes disease. However, despite over a century of investigation into GBS pathogenesis, only a small fraction of the genome has been characterized, with the majority of open reading frames still annotated as “uncharacterized,” or ascribed a putative function on the basis of sequence homology to known proteins in other species. Characterizing proteins of unknown function, especially those that are localized to the bacterial surface, is critical for identifying novel anti-virulence targets or candidate vaccine antigens (Doro et al., 2009; Patras et al., 2018).

We recently pioneered and applied a host membrane-specific virulence determinant enrichment strategy termed Biomimetic Virulomics (BV) to identify and then characterize a novel virulence factor of previously unknown function in group A *Streptococcus* (GAS) (Lapek et al., 2017). This protein was named “S protein,” due to the wide distribution of homologs in the *Streptococcus* genus, and its corresponding open reading frame designated *ess* (Wierzbicki et al., 2019). Functional studies of S protein demonstrated a central role in bacterial physiology, where it impacted the bacterium’s ability to resist killing by components of the host immune response *in vitro* and *in vivo.* We further linked GAS S protein to a novel strategy for evading host immunity, wherein the bacterium coats itself in lysed red blood cell components, preventing the host from recognizing bacterial pathogen-associated molecular patterns, thus contributing to bacterial *in vivo* survival and virulence. Given the important role S protein plays in GAS pathogenesis, we hypothesized that an S protein homolog in GBS would similarly be indispensable for pathogenesis.

Here, we describe a role for S protein in GBS virulence, linking S protein deletion (Δ*ess*) to reduced capsular retention and a destabilized bacterial surface virulome. We also determined that *ess* deletion reduced bacterial surface glycosylation through mass spectrometry-based methods. *In vivo*, the Δ*ess* mutant showed attenuated virulence and increased susceptibility to rapid bacterial clearance. Proteome analysis of blood and spleen tissue collected from infected animals revealed that Δ*ess* elicited increased proteasome components compared to infection with the wild-type (WT) GBS strain, linking rapid clearance of the mutant to increased intracellular proteolysis. Together, these findings implicate the S protein of GBS as a key virulence determinant, resembling its GAS counterpart.

## Materials and Methods

### Ethics Statement

Animal studies were conducted under protocols approved by the UC San Diego Institutional Animal Care and Use Committee (IACUC). Fresh whole blood and blood for neutrophil isolation were obtained via venipuncture from healthy volunteers under written informed consent approved by the UC San Diego Human Research Protection Program.

### Bacterial Culture Methods

Bacteria were grown in sterile Todd-Hewitt broth (THB, Hardy Diagnostics, Santa Maria, CA, United States). WT + pDC*erm*, Δess + pDC*erm*, and Δess + pDC*erm:ess* GBS strains were grown in THB supplemented with 5 μg/mL erythromycin (Thermo Fisher Scientific, Waltham, MA, United States) or Tryptic Soy Agar (TSA) with 5% sheep’s blood (BD BBL, Franklin Lakes, NJ, United States).

### Group B *Streptococcus* S Protein Sequence Analysis

The S protein sequence from the serotype V CNCTC 10/84 strain was subjected to PSI-Blast against the Streptococcus genus (taxid:1301). Matches with > 60% sequence identity and > 70% query coverage were aligned on NCBI MSA viewers. These sequences were used to create a circular dendrogram using the “ggtree” R package. The CNCTC 10/84 S protein coding gene sequence was subjected to BLAST analysis against all complete *Streptococcus agalactiae* genomes on NCBI and resultant matches were aligned and visualized using the “ggmsa” R package. A protein schematic was drawn using the “drawProteins” Bioconductor R package with protein features acquired from UniProt. S protein gene locus mapping was visualized using the “gggnes” R package with gene information acquired from the CNCTC 10/84 complete genome (Ref:NZ_CP006910.1) acquired from NCBI genome.

### Group B *Streptococcus* Mutant Strain Generation

Cloning methods were adapted from multiple sources (Framson et al., 1997; Patras et al., 2018; Wierzbicki et al., 2019). Briefly, the *ess* homolog from GBS was identified in the genome of the well characterized GBS serotype V strain CNCTC 10/84 strain (American Type Culture Collection catalog no. 49447). Genomic DNA was extracted from GBS NCTC 10/84 grown overnight in 10 mL of THB using the Wizard Genomic DNA Isolation Kit using manufacture protocols (Promega, Madison, WI, United States). Isolated genomic DNA was used for subsequent amplification of the *ess* gene locus.

Amplification of the gene-coding region containing the flanking regions of the *ess* homolog was performed using PCR, with forward and reverse primers used to amplify the 500 bp upstream (FW: 5′-AATCCTCCCCGACTTCCCCC TTGTTAATC-3′; REV: 5′-TAGGATTGTATCTTTTAACTTTT TAAG-3′) and downstream (FW: 5′-TTTCTTGATTTTCTT TAAAGCG-3′; REV: ′-GGGGGAAGTCGGGGAGGATTATGAA CTC-3′) of the gene. Q5 High Fidelity Polymerase (New England Biolabs, Ipswich, MA, United States) was used for amplification and confirmed by polyacrylamide gel electrophoresis. Next, Gibson assembly was performed on the fragments by mixing 0.2 pmol of each at a 1:1 ratio along with 10 μL Gibson Assembly Master Mix (New England Biolabs, Ipswich, MA, United States) and 10 μL deionized water. Mixtures were incubated at 50°C for 30 min. The resulting assembly fragments were amplified with Q5 polymerase to include restriction enzyme sites on either side, Xho1 and Not1 (FW: 5′-TATATACTCGAGTTTCTTGATTTTCTTTAAAGCG-3′; REV: 5′-TATATAGCGGCCGCTAGGATTGTATCTTTTAACTTTTT AAG-3′). The fragment and the pHY304 vector were next subjected to enzymatic digestion with Xho1 and Not1 in CutSmart Buffer (New England Biolabs, Ipswich, MA, United States). After digestion, fragments were purified and quantified by NanoDrop. Digestion fragments were ligated together with Quick Ligase (New England Biolabs, Ipswich, MA, United States), transformed into chemically competent DH5α *E. coli* and clones selected on LB (BD Difco, Franklin Lakes, NJ, United States) agar with 500 μg/mL erythromycin to identify cloned vector, and named pAC1. pAC1 identity was checked using PCR for the ligation sites (FW: 5′-GCAAGGCGATTAAGTTGGGT-3′; REV: 5′-GTGTGGAATTGTGAGCGG-3′). Electrocompetent GBS were generated by growing bacteria in 0.6% glycine (MP Biomedical, Irvine, CA, United States) in THB. Bacteria were washed in 0.625M sucrose (Hoefer Inc., Holliston, MA, United States) pH 4.5 and resuspended in an identical buffer (Framson et al., 1997). Purified pAC1 plasmids were mixed with electrocompetent GBS, with 1 μL of pAC1 and 75 μL of bacteria. Samples were gently mixed and incubated on ice for 30 min. Electroporation occurred using 0.1 cm cuvette (Bio-Rad, Hercules, CA, United States) and the following settings: 600 Ω, 1.25 kV, and 25 μF. After electroporation, bacteria were incubated in THB with 0.25 M sucrose for 2 h on a rotator at 30°C. Antibiotic selection was performed on THB with 5 μg/mL erythromycin at 30°C overnight. Positive colonies were shifted to 37°C with erythromycin pressure maintained to allow for single crossover insertion. Cultures were plated on THB media and screened for loss of erythromycin resistance. Erythromycin sensitive strains were expanded in THB media, and genomic DNA extracted. Loss of *ess* (Δ*ess*) or reinstatement of the native gene (revertant) was confirmed by PCR amplification of the gene region and flanking areas (FW: 5′-CATGACTAATTCTTCATGTC-3′; REV: 5′-GGACGTTTTGAATTCGTTAG-3′). The amplified region was then sequenced by Eton Biosciences (San Diego, CA, United States) to ensure that gene knockouts were properly constructed.

For generation of the complemented strain, the Δ*ess* S protein deletion strain of CNCTC in GBS was rendered electrocompetent (with initial growth conditions adjusted to 0.4% glycine). A complementation vector with *ess*, 500 bp upstream, and 327 bp downstream of the chromosomal *ess* gene in the multiple cloning site of the pDC*erm* plasmid was synthesized *de novo* by GenScript (Piscataway, NJ, United States). Electroporation to introduce the plasmid into the Δ*ess* genotype background was performed as described above. In parallel, electrocompetent WT CNCTC and Δ*ess* CNCTC were electroporated in the presence of pDC*erm* lacking components of *ess* to ensure that identical growth conditions could be applied to all strains. Positive clones of all colonies were selected for on THB agar with 5 μg/mL erythromycin. Positive clones of the complemented strain were amplified in liquid media, prepared for plasmid purification, and plasmids were sequenced to confirm introduction of the appropriate plasmid by Eton Biosciences (San Diego, CA, United States).

### Group B *Streptococcus* Growth Rate Analysis

To determine growth rate of GBS strains, overnight cultures were diluted 1:20 in 10 mL of fresh THB media. Samples were grown in triplicate for each strain for 7 h at 37°C. Cultures were resuspended prior to each time point reading and optical density measured at 600 nm (OD_600_). Each sample was serially diluted (10^–1^–10^–5^) with PBS and 5 μL of each dilution was spotted on THB agar for CFU enumeration.

### Hydrophobicity Assay

Bacterial cultures were grown overnight in THB at 37°C. Two mL each of overnight cultures were subjected to centrifugation at 8,000 × g for 2 min. Pellets were washed in sterile PBS twice. Hydrophobicity assay was performed by resuspending bacteria in 2 mL of sterile PBS in borosilicate test tubes. 500 μL of *n*-hexadecane (MP Biomedical) was layered on top of each sample. Negative control duplicates containing only PBS with culture pellets were also prepared. Tubes were covered with parafilm and subjected to vigorous vortexing for 15 s each. Aqueous and organic layers were allowed to separate for 5 min. Hydrophobicity was assessed by measuring OD_600_ of the aqueous fraction and dividing this value by the negative control OD_600_. This number was multiplied by 100 and then subtracted from 100. The experiment was performed in biological triplicate.

### Transmission Electron Microscopy and Capsule Thickness Determination

GBS strains were grown overnight then inoculated 1:10 in fresh THB media. When the culture reached optimal density OD_600_ = 0.4, 1 mL of the culture was spun down, washed once with PBS and was fixed by adding modified Karnovsky’s fixative (2.5% glutaraldehyde + 2% paraformaldehyde in 0.15 M sodium cacodylate buffer, pH 7.4) for at least 4 h, post-fixed in 1% osmium tetroxide in 0.15 M cacodylate buffer for 1 h, and stained in block in 2% uranyl acetate for 1 h. Samples were dehydrated in ethanol, embedded in Durcupan epoxy resin (Sigma-Aldrich, St. Louis, MO, United States), sectioned at 50–60 nm on a Leica 6 UCT ultramicrotome, and picked up on Formvar and carbon-coated copper grids. Sections were stained with 2% uranyl acetate for 5 min and Sato’s lead stain for 1 min. Grids were viewed using a JEOL 1200 EX II TEM transmission electron microscope and images obtained with Gatan 792 and Gatan Orius 600 digital camera. Images were taken from multiple random fields at magnifications ranging from 10,000 × to 50,000 ×. GBS capsule thickness of at least 10 random bacteria from at least 5 random pictures for each strain was measured using the Image J software.

### Supernatant Quantification of Capsule

Capsule quantification was performed using a modified published method (Jin and Pancholi, 2006). Briefly, 5 mL of overnight bacterial cultures were subjected to centrifugation at 8,000 × g for 5 min. Supernatants were segregated from pellets and filtered through 0.22 μm barriers (Millipore Sigma, Burlington, MA, United States). 2 mL of each supernatant (from WT, S protein deletion mutant, and complemented strains) was mixed with 2 mL of a chromogenic reagent comprised of 20 mg of 3,3′-Diethyl-9-methyl-4,5,4′,5′-diben zothiacarbocyanine, 1-Ethyl-2-3-(1-ethylnaphtho1,2-dthiazolin-2-ylidene)-2-methylpropenylnaphtho1,2-dthiazolium bromide (Thermo Fisher Scientific) with 60 μL of glacial acetic acid in 100 mL of 50% formamide in borosilicate test tubes. A standard capsule control was generated using *Streptococcus equi* capsule (Alfa Aesar, Ward Hill, MA, United States). Tubes were subjected to vigorous vortexing and absorbance read at 640 nm. Absorbance value for experimental samples were calculated as a ratio of the *S. equi* standard.

### Whole Blood Killing Assay

Fresh whole blood was acquired from healthy donors using protocols approved by the UC San Diego Human Research Protections Program. Overnight cultures of GBS strains were inoculated to Todd Hewitt Broth (THB) at a ratio of 1:10. When the culture reached OD_600_ = 0.4, the culture was centrifuged and re-suspended in HBSS (Life Technologies, Carlsbad, CA, United States). 100 μL of fresh blood was seeded into a 96 well flat bottom plate. 20 μL of bacteria (5 × 10^4^ CFU/well) prepared above were added on top of seeded blood and incubated for 30 min at 37°C on a horizontal rotor. Bacterial inoculum was reserved for plating to determine whole blood killing. After incubation, water was added to lyse red blood cells. Wells were properly mixed and diluted and plated on to agar plates. Each condition was tested in triplicate and the individual experiments were repeated at least 3 times.

### Neutrophil Killing Assay

Neutrophils were isolated from fresh whole blood of healthy donors using protocols approved by the University of California San Diego (UCSD) Human Research Protections Program. The Polymorphprep (Axis-Shield) was used for extraction of neutrophils following the manufacturer’s protocol. All GBS strains were grown to mid-logarithmic growth phase (OD_60__0__*n*__*m*_ = 0.4) and washed in PBS. Neutrophils were added to bacteria at a multiplicity of infection (MOI) = 0.1 bacteria per neutrophil, centrifuged at 500 × g for 5 min to ensure contact, and incubated for 30 min at 37°C with 5% CO_2_. Prepped neutrophils were re-suspended in HBSS (Ca^+^, Mg^+^ before the assay) at a concentration of 5 × 10^6^ cells/mL. Next, 100 μL neutrophils (5 × 10^5^ cells) were seeded to 96 well flat bottom plate to have each condition in triplicate and 100 μL of bacteria (5 × 10^4^ CFU/well) prepared above was added to each well of neutrophils. The 96 well plate was centrifuged at 500 × g for 5 min and then incubated in 37C + 5% CO_2_ for 30 min. At experiment termination, samples were serially diluted in PBS and plated onto THB agar plates for CFU enumeration. Each condition was tested in triplicate and the individual experiments were repeated at least 3 times.

### Primary Human Neutrophil Oxidative Burst Assay

Human neutrophils (1 × 10^7^/mL) were loaded with 20 μM 2,7-dichlorofluorescein diacetate (DCFH-DA, Sigma Aldrich) in Hank’s balanced salt solution (HBSS, Cellgro) without Ca^2+^ and Mg^2+^ and incubated with rotation at 37°C for 20 min. Neutrophils were washed once with PBS and resuspended in HBSS with Ca^2+^ and Mg^2+^ to a density of 1 × 10^6^ cells/well in a white wall 96 well palate (Costar, Princeton, NJ, United States). Multiplicity of infection (MOI) = 1 bacteria per neutrophil was added to each well and was incubated for 30 min at 37°C with 5% CO_2_. Fluorescence intensity at 485 nm excitation/520 nm emission quantified on an Enspire plate reader (Perkin Elmer).

### Animal Studies

The CD1 mice used in this study were obtained from Charles River (Wilmington, MA, United States). For survival studies, 6 × 10^7^ CFU GBS strains were intraperitoneally injected in to 8 weeks old female CD1 mice. Survival of infected mice were monitored every 8 h for 6 days. For CFU enumeration and mass spectrometry experiments, 4.6–4.8 × 10^7^ CFU GBS strains or PBS controls were I.P. injected into 8 weeks old female CD1 mice. Mice were euthanized 24 h after infection and blood, brain, and spleen were harvested and homogenized. Dilution was plated onto Todd-Hewitt agar plates (THA) for CFU enumeration. Part of the samples were kept in −80°C for proteome analysis.

### Group B *Streptococcus* Surfome Mass Spectrometry Sample Preparation

GBS strains (WT + pDC*erm*, Δ*ess* + pDC*erm*, and Δ*ess* + pDC*erm:ess*) were grown overnight from frozen glycerol stocks in Todd-Hewitt broth (THB) at 37°C. GBS cultures were grown the next day at a 1:20 dilution in THB for 3 h. Bacterial cultures were centrifuged at 12,000 × g for 5 min at 4°C and washed three times with sterile phosphate-buffered saline (PBS). After the last wash, pellets were processed for protein digestion via resuspension in 1M urea with 50 mM HEPES and 10 μg of trypsin rotating at 37°C for 40 min. Digested culture samples were centrifuged at 12,000 × *g* for 5 min and supernatant was removed. Isolated peptides were filtered using Millex-GP 0.22 μm polyethersulfone syringe filters. Peptides were subjected to reduction of disulfide bonds via addition of dithiothreitol (DTT, Life Technologies) to a final concentration of 5 μM and incubation at 56°C for 30 min. Reduced disulfide bonds were alkylated via addition of iodoacetamide (IAA, Sigma Aldrich) to a final concentration of 15 μM and incubation at room temperature in a darkened environment. The alkylation reaction was quenched via addition of DTT to a final concentration of 5 μM and incubation at room temperature on a benchtop. Peptides were then desalted using C18 columns using manufacturer’s instructions (Waters, Milford, MA, United States). Desalted peptides were dried under vacuum and analyzed using an Orbitrap Fusion Mass Spectrometer (see section “Mass Spectrometry Methods”).

### Group B *Streptococcus* Culture Supernatant Mass Spectrometry Sample Preparation

GBS strains (WT + pDC*erm*, Δ*ess* + pDC*erm*, and Δ*ess* + pDC*erm:ess*) were grown overnight from frozen glycerol stocks in 10 mL Todd-Hewitt broth (THB) supplemented with 5 μg/mL of erythromycin at 37°C. Overnight cultures for back diluted 1:20 and allowed to grow for 3 h and then spun down at 10,000 × g for 10 min. Culture supernatant were removed and filtered using Millex-GP 0.22 μm polyethersulfone syringe filters. A quarter of a cOmplete, Mini EDTA-free Protease Inhibitor Cocktail tablet (Roche, Basel, Switzerland) was added to each filtered culture supernatant. Reduction of protein disulfide bonds was conducted by addition of 500 mM dithiothreitol (DTT) to a final concentration of 5 mM, vortexed and incubated at 47°C. Alkylation of reduced disulfide bonds was executed by the addition of iodoacetamide (IAA) to a final concentration of 15 mM, and incubated at room temperature, in a darkened environment, The reduction reaction was quenched by the addition of DTT to a final concentration of 5 mM. Protein precipitation and digestion was conducted using chloroform-methanol extraction. Samples were mixed with 4x sample volume of methanol, followed by the addition of 1x sample volume of chloroform, and 3x sample volume of HPLC-grade water. Samples were subsequently centrifuged at 4,000 rpm for 2 min and protein pellets were washed twice with 300 μL of acetone. Protein pellets were dried at 56°C and resuspended in 300 μL of digestion buffer composed of 50 mM HEPES, 1M Urea, and 5 μg of trypsin (Promega, Madison, WI, United States). Trypsin digest was conducted at 37°C for 3 h and quenched through acidification with 20 μL of 10% trifluoroacetic acid (TFA). Peptides were dried through vacuum centrifugation and stored at −80°C. Samples were desalted using C_18_ resin columns (Waters, Ipswich, MA, United States).

Dried samples were resuspended in 50 μL of a solution of 30% dry acetonitrile and 200 mM HEPES (pH 8.5). TMT reagents (Thermo Fisher Scientific) were resuspended in 30% dry acetonitrile and 50 mM HEPES (pH 8.5). Sample labeling was performed by adding 7.5 μL of appropriate TMT label on each sample with an incubation period of 1 h at room temperature. The labeling reaction was quenched using 9 μL of 5% hydroxylamine per sample tube with an incubation period of 15 min.

Multiplexed samples were resuspended in 105 μL of 25 mM ammonium bicarbonate and fractioned on an Ultimate 3000 HPLC using a gradient ranging from 5 to 35% acetonitrile with 10 mM ammonium bicarbonate, wherein 24 fractions were collected over 75 min. Twelve alternating fractions were dried down through vacuum centrifugation and were analyzed using an Orbitrap Fusion Mass Spectrometer (see section “Mass Spectrometry Methods”).

### Organ Proteome Mass Spectrometry Sample Preparation

Lysed organ samples were sonicated using a Q500 QSonica sonicator with a 1.6 mm tip at 20% amplitude for a pulse rate of 10 s on, 10 s off for three cycles. Reduction of protein disulfide bond was performed using 5 μL of 1 M dithiothreitol (DTT). Samples were vortexed and incubated at 47°C for 30 min. Alkylation of reduced disulfide bonds was performed using 15 μL of 1M iodoacetamide (IAA) in a dark environment at room temperature for 45 min. The alkylation reaction was quenched using 5 μL of 1 M DTT.

S-trap mini by ProtiFi (Farmingdale, NY, United States) was used for protein extraction and digestion. Briefly, samples were prepared by addition of 27 μL of 12% phosphoric acid to reduced and alkylated samples. Binding buffer containing 90% methanol and 50 mM TEAB, adjusted to pH 7.1 was added to samples at a 7:1 ratio to sample volume. Samples were loaded on to S-trap mini spin columns. Bound samples were washed with 165 μL of binding buffer. Samples were digested using 5 μL trypsin (2.5 μg) and 115 μL 50 mM TEAB per sample and an incubation period of 3 h at 47°C. Peptides were eluted by using 125 μL 50 mM TEAB, 125 μL 5% FA, and 125 μL 50% ACN, 5% formic acid (FA) in individual subsequent steps. Eluted peptides were dried under vacuum. Dried down peptide pellets were resuspended in 0.1% trifluoroacetic acid (TFA). Samples were then desalted using C_18_ resin columns and again dried under vacuum.

Dried samples were resuspended in 50 μL of a solution of 30% dry acetonitrile and 200 mM HEPES (pH 8.5). TMT reagents (Thermo Fisher Scientific) were resuspended in 30% dry acetonitrile and 50 mM HEPES (pH 8.5). Sample labeling was performed by adding 7.5 μL of appropriate TMT label on each sample with an incubation period of 1 h at room temperature. Labeling reaction was quenched using 9 μL of 5% hydroxylamine per sample tube with an incubation period of 15 min.

Multiplexed samples were fractioned with Pierce High pH Reversed-Phase Peptide Fractionation Kit (Thermo Fisher Scientific) using the manufacturer’s protocol. Briefly, samples were resuspended in 300 μL of 0.1% TFA solution. Samples were bound to the resin and eluted using increasing concentrations of acetonitrile. After fractionation samples were dried using vacuum centrifugation. Eight fractions resulted from each multiplexed experiment, and were analyzed using an Orbitrap Fusion Mass Spectrometer (see section “Mass Spectrometry Methods”).

### Mass Spectrometry Methods

Mass spectrometry-based proteome analysis was performed on an Orbitrap Fusion Mass Spectrometer with in-line Easy nano-LC. The LC was connected to the mass spectrometer via an in-house pulled and packed column with the following characteristics: the column was 30 cm long column with contents starting from the spray tip as follows: 0.5 cm of 5 μm C4, 0.5 cm of 3 μm C18, and 29 cm of 1.8 μm C18. The inner diameter was 100 μm, while the outer was 350 μm. The column, sample injection, and waste lines were connected via a T-junction which was electrified at 2,000 V to induce nanospray ionization.

For surfome proteomics, 12 alternating fractions were resuspended in 8 μL of 5% ACN with 5% FA, and 3 μL of each were injected onto the fractionation column. For culture supernatant proteomics, 12 alternating fractions were resuspended in 10 μL of 5%ACN with 5% FA and 1 μL was used for analysis. For organ proteomics, all 8 resultant fractions were resuspended as above, but only 1 μL of each was used for analysis for blood and spleens, while 3 μL were used for brain samples.

MS1 data were acquired in data-dependent mode using a scan range of 500–1,200 m/z and resolution of 60,000. Maximum inject time was 100 ms and automatic gain control (AGC) was 2 × 10^5^. MS2 data were collected using the decision tree option, with two possibilities: ions with 2 charges were analyzed within the 600–1,200 m/z range, while those with 3–4 charges were acquired between 500 and 1,200 m/z. The lower threshold for ion fragmentation was 5 × 10^4^. Ions selected for fragmentation in the quadropole at 0.5 Th were fragmented using CID in the linear ion trap in centroid mode. Rapid scan rate was used and the AGC setting was 1 × 10^4^. MS3 based quantitation was performed in the Orbitrap following HCD fragmentation. Reporter ion detection occurred with 60,000 resolution and AGC of 1 × 10^5^ with maximum ion inject time of 100 ms.

### Proteome Data Processing

**FIGURE 1:**
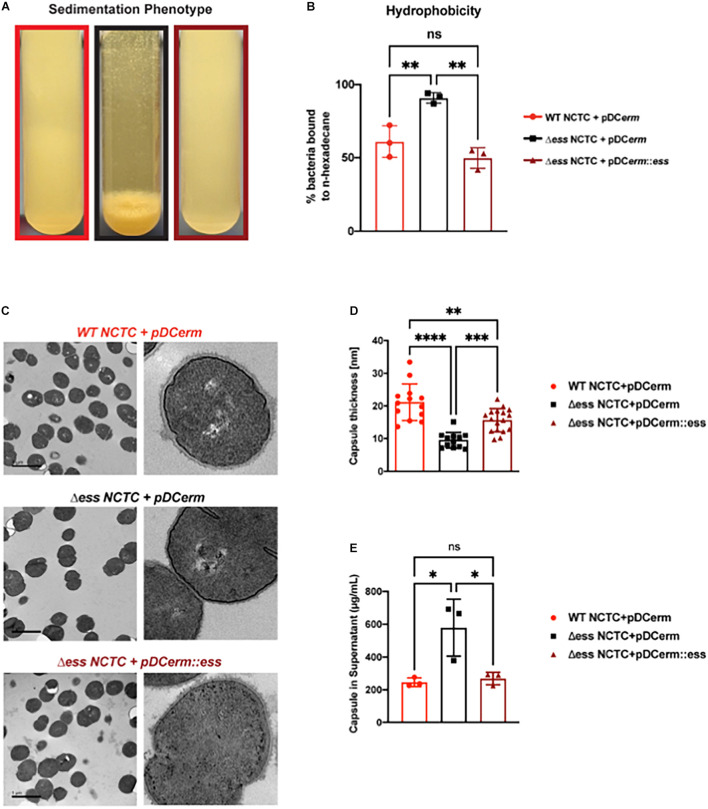
Deletion of S protein alters properties of surface chemistry in GBS. **(A)** Photo demonstrating sedimentation phenotype of WT, S protein deletion, and complemented GBS strains. **(B)** Hydrophobicity assay of WT, S protein deletion, and complemented GBS strains. Significance was determined using Tukey’s Multiple Comparisons Test (^∗∗^*p* < 0.01). **(C)** Transmission electron microscopy of WT, S protein deletion, and complemented GBS strains. Images to the right are representatives of images on the left, blown up to demonstrate cell wall and capsule morphology. **(D)** Capsule thickness quantified in ImageJ. Significance was determined using Tukey’s Multiple Comparisons Test (^∗∗^*p* < 0.01; ^∗∗∗^*p* < 0.001; ^*⁣*⁣**^p < 0.0001). **(E)** Capsule detected in supernatant using Stains-all and normalized against a *Streptococcus equi* capsule standard (^∗^*p* < 0.05).

Proteome data were searched using Proteome Discoverer. Surfome and culture supernatant data were searched against the reference proteome for *Streptococcus agalactiae* serotype V downloaded from Uniprot.com. Organ proteome data was searched against the *Mus musculus* reference proteome downloaded from Uniprot.com. The Sequest algorithm was used to facilitate spectral matching to an *in silico* theoretical database generated from each reference proteome (Eng et al., 1994). The mass tolerance for precursor ions was 50 ppm and the fragment ion mass tolerance was 0.6 Da. Two missed cleavages were allowed, and peptide length was relegated to 6–144 amino acids. Allowed modifications included oxidation of methionine (variable) and TMT labeling of lysines and N-termini and carbamidomethylation of cysteine (static). For glycoproteome analysis of surfome data, a Byonic node replaced the Sequest node, allowed static modifications were expanded to include putative sugar modifications specified in Figure 1E. and A false discovery rate of 1% was applied for filtering for peptides and proteins.

Following completion of each search, proteome data were processed and normalized. For processing, peptide spectral matches (PSMs) were filtered to exclude matches without “High” confidence and with a “Rejected” PSM ambiguity. They were also filtered to retain only those PSMs that had average quantitation abundance > 10 and had isolation interference value < 25. PSMs were summed to the protein level, or for glycoproteome investigations, to the unique modified peptide level. Summed values were normalized to the average value for each unique entity, which were themselves normalized to the median of the averages. The organ proteome data values were next subjected to channel-based normalization, where each value was divided by the median for a specific channel, which was itself divided by the median of all protein abundance values.

### Data Analysis and Figure Generation

For biological assays, one-way ANOVA statistical tests with Tukey’s Multiple Comparison Test was performed using GraphPad Prism. Statistical significance was denoted using the following scheme: ns: non-significant; ^∗^*p*-value < 0.05; ^∗∗^*p*-value < 0.01; ^∗∗∗^*p*-value < 0.001; ^****^p-value < 0.0001. For proteome data where binary comparisons were performed, statistical significance was determined for two comparison groups either using *p*-value based significance metrics or π score, a significance metric incorporating both *p*-value and fold change. For proteomics data, *p*-value was determined using Students *T*-test with Welch’s correction in instances where the assumption of equal variances could not be fulfilled based on *F*-test values.

Heatmap generation for hierarchical clustering was performed in Morpheus.^1^ Venn Diagrams were generated using BioVenn (Hulsen et al., 2008). Cytoscape was used to process String interaction network-based figures. All other graphs were made using GraphPad Prism. All figures were compiled and processed in Adobe Illustrator.

### Data Availability

Proteome data was uploaded to massive.ucsd.edu and can be accessed using the following identifiers: PXD026318 for GBS surfome analysis, PXD026319 for blood proteome analysis, PXD026396 for spleen proteome analysis, PXD026418 for brain proteome analysis, and PXD028628 for culture supernatant analysis.

### Data Availability Statement

The raw data supporting the conclusions of this article will be made available by the authors, without undue reservation.

## Results

### S Protein Is Conserved Among Group B *Streptococcus* Serotypes

**FIGURE 2:**
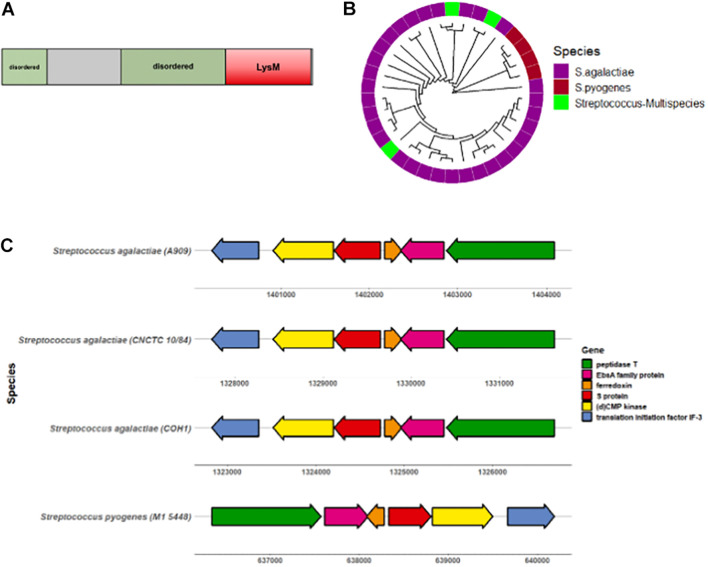
S protein is conserved amongst GBS serotypes. **(A)** Diagram showing GBS S protein functional protein domains. **(B)** Circular dendrogram showing phylogeny of S protein homologs in Streptococcus genus acquired from PSI-BLAST of the CNCTC 10/84 strain S protein sequence. Sequences with > 60% sequence identity and > 70% query coverage for used for phylogeny analysis. **(C)** Gene locus location of GBS S protein (*ess*) and surrounding genes.

To begin characterizing the S protein homolog in GBS, we first sought to determine the degree of conservation among diverse GBS strains. The GBS S protein homolog is an 18.4 kDa protein with a conserved LysM motif and two predicted disordered regions (Figure 2A). The S protein sequence from the virulent serotype V CNCTC 10/84 GBS strain was subjected to PSI-BLAST, of which 29/43 sequences were attributed to *Streptococcus agalactiae* (GBS). These alignment of these sequences was visualized via construction of a circular dendrogram (Figure 2B). To further assess the degree of similarity of GBS S protein, the CNCTC 10/84 strain S protein sequence was subjected to NCBI MegaBLAST against complete annotated GBS genomes. These results showed > 99% similarity of S protein within these genomes (Supplementary Figure 1A). Mapping of the S protein-containing gene region revealed a high level of conservation of flanking genes surrounding S protein in diverse sets of GBS strains as well as with *Streptococcus pyogenes* (GAS) (Figure 2C). Conserved genes included those encoding for peptidase T, an EbsA family protein, ferrodoxin, (d)CMP kinase, and translation initiation factor IF-3.

### Deletion of S Protein Alters Properties of Surface Chemistry in Group B *Streptococcus*

To continue studying the role of the S protein homolog in GBS, we generated an S protein deletion mutant (Δ*ess*) in the CNCTC 10/84 strain (Hooven et al., 2014). A revertant strain (restoring the WT) was collected from the single crossover stage of the *ess* gene deletion procedure. In addition, the Δ*ess* allelic replacement mutant was complemented by transformation with plasmid vector pDC*erm* expressing the cloned *ess* gene. In stationary liquid culture, a sedimentation phenotype was immediately apparent in the Δ*ess* strain, in contrast to the WT strain, which remained dispersed throughout the medium in culture. This phenotype was complemented upon reintroduction of S protein on an exogenous vector (Figure 1A). Previous studies have linked increased bacterial cell sedimentation with changes in cell surface hydrophobicity (Kumar et al., 1991; Zita and Hermansson, 2006; Araújo et al., 2008; Krasowska and Sigler, 2014). Therefore, we tested whether the Δ*ess* strain showed altered hydrophobicity by evaluating its differential propensity to interact with aqueous vs. organic solvents (Figure 1B). As hypothesized, Δ*ess* associated more readily with the organic solvent compared to the WT or complemented strains, suggesting that the bacterial aggregation phenotype was caused by increased surface hydrophobicity driving bacterial self-association.

The best characterized GBS virulence determinant is its capsule, a layer of polysaccharide coating the cell surface that allows bacteria to evade clearance by host innate immunity (Cieslewicz et al., 2005). Encapsulation of GBS renders the bacteria hydrophilic, due to the polar nature of sugars. Given our finding demonstrating increased hydrophobicity in Δ*ess*, we sought to determine if the altered surface character in the mutant strain was due to a defect in encapsulation. Transmission electron microscopy (TEM) revealed altered capsular morphology with a reduced capsule thickness and relatively smoothed appearance in the Δ*ess* mutant in comparison to the WT or complemented strains (Figures 1C,D). Reduced capsule thickness in the Δ*ess* mutant was accompanied by higher levels of sugars in the supernatant of bacterial cultures in Δ*ess* compared to the WT or complemented strains, suggesting diminished capsule retention in the absence of S protein (Figure 1E). Together, these data suggest that S protein plays an important role in GBS surface architecture and in particular the surface presentation of GBS capsule.

GBS pigment and β-hemolysin/cytolysin (β-H/C) are well-established virulence factors leading to hallmark phenotypes (Whidbey et al., 2013; Rosa-Fraile et al., 2014) often indicative of the degree of virulence in distinct strains. As the deletion of S protein showed distinct surface chemistry, we sought to uncover any differences in pigmentation and hemolytic capabilities of the Δ*ess* and complemented strains in comparison to WT. We found the Δ*ess* strain demonstrated lack of pigmentation in comparison to WT and complemented strains but retained hemolytic capabilities (Supplementary Figure 2A). Furthermore, growth curve analysis of all three strains demonstrated diminished levels of colony forming units (CFU) over time in comparison to WT and complemented strains (Supplementary Figures 2B,C). These results further cement the role of S protein in bacterial cell surface integrity.

### Group B *Streptococcus* S Protein Stabilizes the Surface-Anchored Virulome

Proteins exposed on the bacterial surface play a critical role in mediating bacterial interactions with the environment, including the relationship of pathogens with host defenses. We next explored potential changes in surface-associated protein dynamics resulting from loss of S protein expression. Enzymatic proteolytic surface shaving was paired with quantitative proteomic analysis of the WT, Δ*ess*, and complemented strains (Rodríguez-Ortega et al., 2006; Doro et al., 2009), adapting a strategy used in the past to profile surface-exposed epitopes for GBS vaccine development.

**FIGURE 3:**
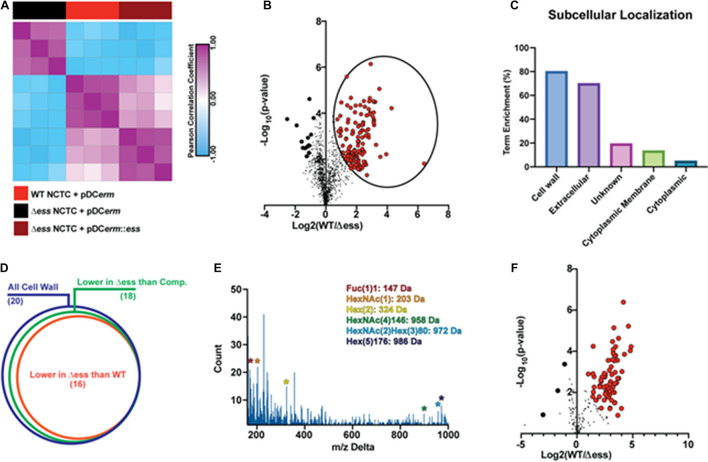
GBS S protein stabilizes the surface-anchored virulome. **(A)** Similarity matrix demonstrating Pearson correlation values for proteome quantitation data following hierarchical clustering. **(B)** Binary comparison strategy for identifying differentially abundant proteins in the surfome samples collected from WT and S protein deletion mutant GBS strains. Proteins significantly higher in WT compared to the deletion mutant are circled. Proteins with π score value greater than 2.5 were highlighted for further analysis. **(C)** Results of pSORTb analysis of proteins significantly upregulated in WT GBS in comparison to the S protein deletion mutant. Data are reported as the percentage of significantly increased proteins in a given location within the total number proteins assigned a location. **(D)** Venn diagram showing proportion of all cell wall proteins identified upregulated in WT or complemented strains compared to the S protein deletion strain as a proportion of all cell wall proteins. **(E)** Delta m/z histogram of PTM-inclusive search of surfome data with putative glycans highlighted. **(F)** Binary comparison of glycosylation-inclusive data for WT vs. S protein deletion surfome data. Glycopeptides with differential abundance π score greater than 2.5 are highlighted.

Surface shaving of bacterial cells was performed in biological triplicate, and quantitative proteomic analysis carried out using tandem mass tags (TMTs), which allowed for multiplexing of the samples prior to mass spectrometry-based analysis. From the 12 fractions analyzed, 1,212 proteins were identified and quantified after quality control filtering. Unbiased hierarchical clustering was performed on the proteome data to evaluate the similarities and differences within the data from a broad perspective. Hierarchical clustering showed that the Δ*ess* mutant segregated from the WT and complemented strains. WT and complemented strains were correlated, though not to the degree of within-strain replicates (Figure 3A). These data demonstrate that removal of S protein broadly remodels the surface proteome of GBS.

To evaluate the molecular changes in the bacterial surfome associated with loss of S protein, we performed a binary comparison of protein-level quantitative information between the WT and Δ*ess* strains (Figure 3B). Of particular interest to this analysis were those protein abundance values that were higher in the WT strain compared to Δ*ess* (circled in Figure 3B). Among the total proteins identified were a number of putative intracellular contaminants, including ribosomal proteins likely released into the supernatant from lysed cells (Supplementary File 1). Proteins downregulated in the Δ*ess* mutant compared to the WT strain were subjected to pSORTb analysis to evaluate their predicted cellular localization within the bacteria (Yu et al., 2010). Compared to all cell wall-associated proteins identified, 80% were significantly higher in the WT compared to the Δ*ess* surfome. Following this trend, among all extracellular proteins identified, 70% were significantly higher in the WT strain compared to Δ*ess* (Figure 3C). The trend of cell wall-associated proteins being reduced in the absence of S protein was preserved in a comparison of the complemented strain with Δ*ess*, indicating that S protein plays a significant role in stabilizing the surfome (Figure 3D and Supplementary Figures 3A–P). Though the majority of cell wall-associated proteins identified through this analysis remain incompletely characterized, many bear homology to known GBS virulence factors. Among these proteins was SAG0771, a predicted StrA subfamily protein containing an LPXTG motif (Supplementary Figure 3A). Also identified was SAG0416, a protein similar to the C5a peptidase expressed by many virulent *Streptococci* (Supplementary Figure 3K).

Protein modifications also play important roles in overall chemical traits of biological surfaces, and we assessed whether such modifications, especially glycosylation, were altered in the Δ*ess* mutant. Due to the diverse nature of these modifications, we used an unbiased strategy to identify possible glycosylation modifications present in the surfome data. By spectral networking we matched prominent m/z differences between networked spectral features of our data to known glycosylation modifications (Figure 3E; Wang et al., 2016; Wozniak et al., 2020). We then re-searched our data using parameters related to these mass differences. In a focused evaluation of modified peptides, we found that glycosylated peptides were present in lower abundance in the Δ*ess* mutant compared to the WT strain (Figure 3F). Collectively, these findings demonstrate that removal of S protein severely impacts the overall molecular nature of the GBS surfome.

### Absence of S Protein Alters Extracellular Proteome

**FIGURE 4:**
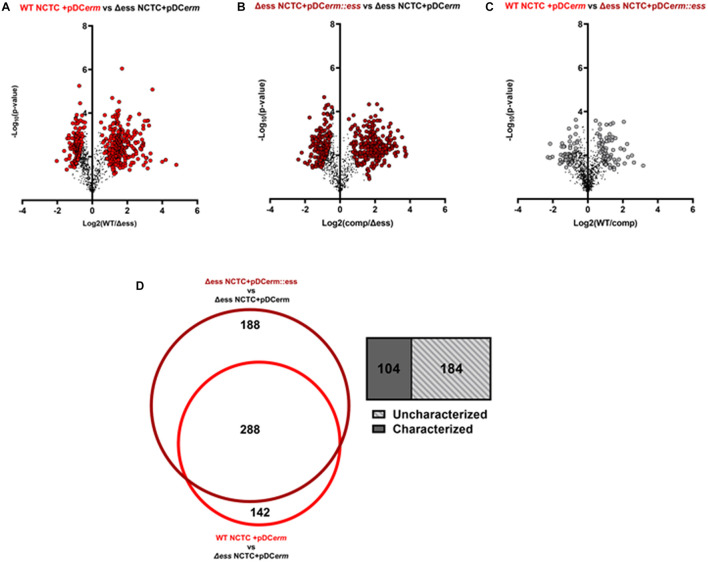
**(A)** Volcano plots demonstrating a binary comparison of differentially abundant proteins in GBS culture supernatant of WT and S protein deletion strains. Proteins with pi-score > 1.5 are shown in bright red. **(B)** Volcano plots demonstrating a binary comparison of differentially abundant proteins in GBS culture supernatant of complemented and S protein deletion strains. Proteins with pi-score >1.5 are shown in dark red. **(C)** Volcano plots demonstrating a binary comparison of differentially abundant proteins in GBS culture supernatant of complemented and S protein deletion strains. Proteins with pi-score > 1.5 are shown in gray. **(D)** Venn diagram showing overlap of differentially abundant proteins when making binary comparison of WT and complemented strains with S protein deletion strain. Box-plot demonstrating proportion of overlapping proteins determined to be uncharacterized on UniProt.

As the deletion of S protein led to altered surface chemistry, we performed quantitative proteome analysis of culture supernatants collected from WT, Δ*ess*, and complemented GBS strains to comprehend changes to the extracellular proteome. Culture supernatant experiments revealed 429 differentially abundant proteins (π-score > 1.5) between WT and Δ*ess*, 475 between complemented and Δ*ess*, and 126 between WT and complemented strains (Figure 4A–C). Proteins that were similarly dysregulated in Δ*ess* and complemented strains in comparison to WT were removed from further analysis. A core set of 288 proteins were determined to be differentially expressed after S protein deletion, of which 184 are uncharacterized (Figure 4D).

Among the upregulated proteins in the deletion strain were two DNA-response regulators—CiaR and CsrR (CovR) (Supplementary Figure 4). CiaR has been shown to be important in GBS stress tolerance and intracellular survival within immune and endothelial cells (Quach et al., 2009). CsrR is a two-component system in a variety of bacterial species (Lamy et al., 2004). In GBS, CovR has been regarded as a master regulator of various virulence factors, namely CAMP factor and the GBS β-hemolysin/cytolysin (β-H/C) (Zhu et al., 2020) as well regulation host cell infiltration (Lembo et al., 2010; Gendrin et al., 2018). Downregulated proteins in the deletion strain included the major sortase enzyme SrtA (Lalioui et al., 2005) as well as the capsular polysaccharide biosynthesis protein, C which are both vital in maintaining bacterial cell surface integrity (Supplementary Figure 4). There was in overall trend in differential abundance for proteins required for membrane transport, lipoproteins, proteases, and kinases, a trend which has been previously described in proteomic and transcriptional analyses of instances of increased invasiveness for GBS (Johri et al., 2007). Altogether, these results indicate S protein deletion leads to alteration of extracellular GBS components which can alter host and bacterial cell interactions.

### S Protein Deletion Sensitizes Group B *Streptococcus* to Killing *in vitro* and *in vivo*

**FIGURE 5:**
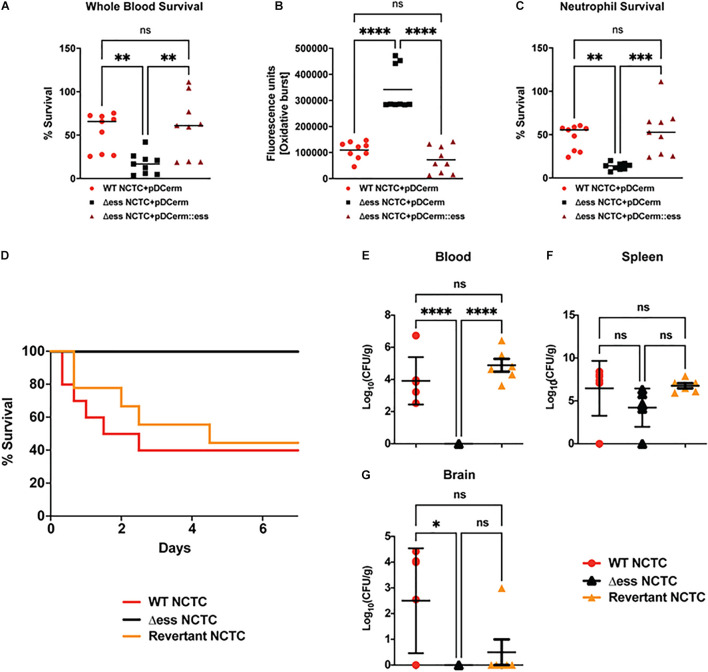
GBS S protein is critical for GBS virulence *in vitro* and *in vivo.* **(A)** Whole blood survival assay results. Significance was determined using Tukey’s Multiple Comparisons Test (^∗∗^*p* < 0.01). **(B)** Neutrophil oxidative burst assay results. Significance was determined using Tukey’s Multiple Comparisons Test (^*⁣*⁣**^p < 0.0001). **(C)** Bacterial survival following incubation with primary human neutrophils. Significance was determined using Tukey’s Multiple Comparisons Test (^∗∗^*p* < 0.01; ^∗∗∗^*p* < 0.001). **(D)** Kaplan-Meier plot demonstrating survival after I.P. challenge with GBS strains. **(E)** Bacterial CFUs recovered from blood 22 h post I.P. infection with GBS strains. Significance was determined using Tukey’s Multiple Comparisons Test (^*⁣*⁣**^p < 0.0001). **(F)** Bacterial CFUs recovered from spleen 22 h post I.P. infection with GBS strains. **(G)** Bacterial CFUs recovered from brains 22 h post I.P. infection with GBS strains. Significance was determined using Tukey’s Multiple Comparisons Test (^∗^*p* < 0.05).

Because loss of S protein was associated with altered capsular retention and surface-associated virulence stability, we examined whether S protein deletion impaired GBS resistance to clearance by host immune defenses in the blood. The Δ*ess* mutant had significantly reduced survival in freshly-isolated whole human blood compared to the WT and complemented strains (Figure 5A). Neutrophils are the most abundant circulating leukocyte and occupy a central role in bloodstream innate immune defense against GBS. The pathogen produces an array of surface-anchored virulence factors that allow the bacterium to evade neutrophil killing (Liu et al., 2004). Neutrophil reactive oxygen species (ROS) release was assayed following to exposure GBS test strains. The Δ*ess* strain elicited significantly higher ROS production compared to WT or complemented strains, consistent with the ability of the GBS capsule that cloaks proinflammatory cell wall components (Figure 5B). Consistent with this finding, loss of S protein resulted in more efficient killing of the Δ*ess* strain by human neutrophils (Figure 5C).

Next, we examined whether the reduced survival of the Δ*ess* mutant in whole blood and results of the neutrophil killing assays translated into reduced virulence during systemic infection *in vivo*. Cohorts of mice were injected intraperitoneally (I.P.) with WT and Δ*ess* GBS strains. As our prior studies using a complementation vector to assess the role of S protein in GAS virulence *in vivo* revealed poor plasmid retention in the absence of antibiotic pressure (Wierzbicki et al., 2019), we added the GBS revertant in to the analysis in lieu of a complemented strain (Patras et al., 2018). Survival was assessed for 7 days after I.P. infection, during which time 60% of animals infected with the WT and revertant strains succumbed to infection. In contrast, 100% of animals infected with the Δ*ess* mutant survived indicating that S protein plays an important role in mediating virulence during GBS infection *in vivo* (Figure 5D).

To gain a deeper understanding of the role S protein plays in the host-GBS interaction *in vivo*, we performed additional studies to evaluate dissemination of GBS into the organs of infected animals. Animals were infected with WT, Δ*ess*, and revertant strains of GBS and sacrificed 8 h post infection. Tissues collected for CFU enumeration included blood, spleen, and brain, the latter chosen due to the propensity of GBS to invade the central nervous system and cause meningitis. Whereas the Δ*ess* mutant was completely cleared from the blood of the infected animals, high levels of blood CFUs were detected for the WT and revertant strains (Figure 5E). In contrast to blood, spleens showed no significant difference in bacterial levels or comparative size (Figure 5F and Supplementary Figure 5A). The spleen plays an important role in filtering the blood and relaying active immune signals (Lewis et al., 2019). Though bacterial levels in the spleens of infected animals did not differ by strain, host pathways engaged by each strain may differ, especially given the rapid clearance of Δ*ess* from the blood. CFU enumeration of recovered brains revealed significantly lower bacterial levels in the Δ*ess* mutant strain-infected mice compared to the WT strain, with the revertant strain yielding an intermediate phenotype (Figure 5G) that did not reach statistical significance (Doran et al., 2005; van Sorge et al., 2009; Banerjee et al., 2011). Overall, these data are consistent with a role for S protein in modulating resistance to immune clearance in the bloodstream and potentially facilitating its further dissemination to the brain tissues.

### S Protein Deletion Mutant Elicits Altered Host Signaling in Key Organs Associated With Clinical Manifestations of Group B *Streptococcus*

**FIGURE 6:**
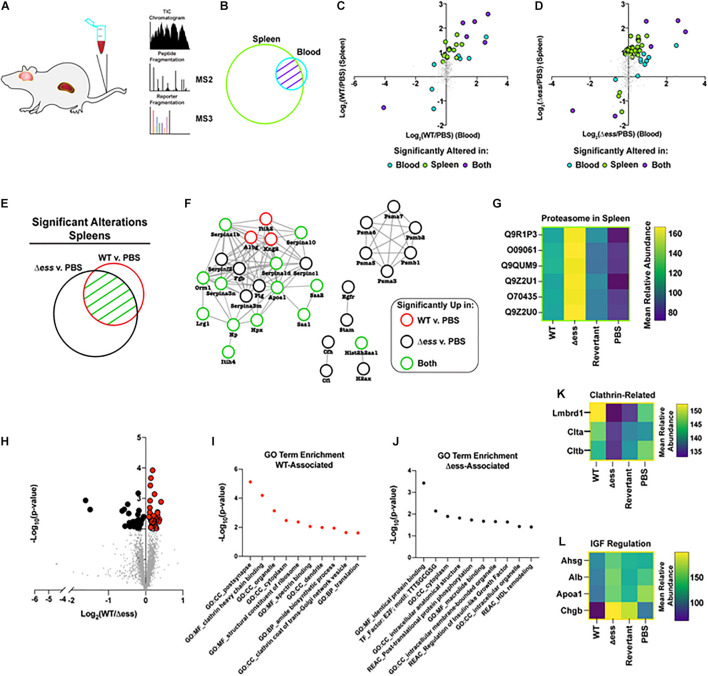
Proteome-based evaluation of S protein-dependent host responses. **(A)** Schematic of mouse organ proteome experiment. **(B)** Venn diagram demonstrating overlap in proteins detected and quantified in blood and spleens following proteome analysis. **(C)** Scatterplot demonstrating Log_2_ fold change of WT infected vs. uninfected GBS strains for blood and spleen proteome data. Highlighted datapoints represent significantly altered proteins (π score > 1.5). **(D)** Scatterplot demonstrating Log_2_ fold change of S protein deletion mutant infected vs. uninfected GBS strains for blood and spleen proteome data. Highlighted datapoints represent significantly altered proteins (π score > 1.5). **(E)** Venn diagram demonstrating significantly altered proteins in spleen comparison of WT or S protein deletion mutant strain compared to uninfected. **(F)** Protein interaction network for the union of significantly altered spleen proteins in comparison of WT or S protein deletion mutant strain vs. uninfected. **(G)** Heatmap demonstrating proteasome components upregulated in spleens of animals infected with S protein deletion mutant compared to WT GBS infected or uninfected spleens. **(H)** Volcano plot demonstrating significantly altered (*p* < 0.01) proteins in the comparison of brains collected from WT vs. S protein deletion mutant GBS following normalization against uninfected. **(I)** Non-redundant top enriched functional terms from proteins significantly increased in brains of mice infected with WT GBS compared to the S protein deletion mutant. **(J)** Non-redundant top enriched functional terms from proteins significantly increased in brains of mice infected with S protein deletion mutant compared to the WT strain. **(K)** Mean relative abundance heatmap of clathrin-related proteins significantly upregulated in WT GBS infected brains compared to S protein deletion mutant infected brains. **(L)** Mean relative abundance heatmap of Insulin-like Growth Factor regulation-related proteins significantly upregulated in WT GBS infected brains compared to S protein deletion mutant infected brains.

Due to the strain-dependent levels of bacterial CFUs recovered from the blood of infected animals, we hypothesized that these differences may be reflected in divergent proteome patterns at the organ level. To address this, we prepared collected organ lysates for quantitative multiplexed proteome analysis (Figure 6A). Following proteome analysis and data normalization, the blood dataset was found to be comprised of 546 proteins, the spleen dataset of 3,384 proteins, and the brain dataset was made up of 3,304 proteins. Due to the close functional relationship between the blood and spleen, we endeavored to investigate the systemic alterations for proteins identified in both tissues (Figure 6B).

Of particular interest within this dataset were proteins with altered abundance in the blood compared to the spleens during infection with either the WT or Δ*ess* GBS strains. To visualize such protein abundance trends, the Log_2_ fold change of the infected vs. uninfected groups were plotted, comparing the spleens and the blood. Proteins possessing a π score greater than 1.5 were highlighted, and were colored to delineate proteins that were significantly altered in the blood alone, the spleens alone, or in both (Xiao et al., 2014). A clear trend that emerged from this analysis was the increased number of significantly altered proteins for the S protein deletion mutant-infected animals (51 proteins) compared to the WT GBS infected animals (27 proteins) (Figures 6C,D). The majority of dysregulated proteins were increased in abundance in both the blood and spleens, though rarely to a degree that met the applied significance threshold in both organs. This data trend, paired with the rapid clearance of the Δ*ess* mutant from the blood, suggested that the protein alterations during infection with the mutant strain may play a contributing role in the enhanced survival for these animals.

We next focused our analysis on the spleens of infected animals to evaluate the differential immune responses associated with rapid clearance of Δ*ess* in comparison to WT infection. The union of proteins identified with significantly altered abundance in the WT and Δ*ess* strains compared to uninfected spleens were used for this analysis (Figure 6E). These proteins were subjected to analysis with String-db, an analytical tool that generates interaction networks on the basis of experimentally demonstrated and predicted protein pathway relationships (Figure 6F). The nodes were colored to indicate whether proteins were significantly altered in the spleens of animals infected with the WT strain, the Δ*ess* mutant, or both strains in comparison to uninfected spleens. A subset of acute phase reactants was significantly increased in response to both infection scenarios, including serine protease inhibitors, Serpinas 3n/1d/c1/10/1b, all known to play a regulatory role the immune response to bacterial infections. Among networked proteins, the great majority were altered in either both infection states or only in the Δ*ess* mutant infection. Only three proteins, Itih2, A1bg, and Kng2, were significantly increased during infection with the WT strain only. One notable set of proteins exclusively increased in the spleens of animals infected with Δ*ess* was Psma1/3/4/5/6/7 (Figure 6G), which comprise an important subunit of the proteasome, an intracellular protein complex that proteolytically cleaves foreign antigens (Kovacsovics-Bankowski and Rock, 1995; Reis e Sousa and Germain, 1995). Proteasomal activation is necessary for the clearance of several known viral, bacterial, and parasitic infectious agents (Strehl et al., 2006; Basler et al., 2009; Joeris et al., 2012; Iovino et al., 2014; Mundt et al., 2016). Enhanced susceptibility to proteasomal activation, therefore, could be a contributing factor in the rapid *in vivo* clearance of the Δ*ess* mutant.

To further evaluate the role of S protein in GBS infection of brain tissues, we performed a binary comparison between the brain proteome data from WT GBS infected animals and Δ*ess* infected animals (Figure 6H). Proteins with differential abundance that met the applied threshold of *p*-value < 0.01 were highlighted for further study, and gene ontology analysis performed on the set of proteins increased during infection with the WT strain compared to Δ*ess* (Figure 6I). Among the top enriched proteins identified in this dataset were clathrin-related proteins (Figure 6K; Mu et al., 2016; Herold et al., 2019). Among proteins with reduced abundance in the brains of animals infected with WT GBS compared to Δ*ess* were several associated with high density lipoprotein (HDL) remodeling (Figure 6J) such as were Ahsg, Alb, ApoA1, and Chgb, proteins with still unelucidated roles in brain health and disease (Figure 6L).

## Discussion

GBS remains an important human pathogen and a significant cause of poor clinical outcomes for afflicted neonates (Libster et al., 2012). A more complete understanding of GBS virulence processes is needed to advance novel therapeutics or preventative strategies against this pathogen. Here, we characterized the GBS S protein homolog to a virulence determinant originally identified in GAS. The data presented demonstrate that the GBS S protein plays a role in the ability of GBS to maintain its surface homeostasis and to resist bloodstream clearance. GBS S protein sequence and gene locus are highly conserved within diverse sets of GBS strains. As with S protein in GAS, deletion of S protein in GBS results in alterations to bacterial physiology and reduced virulence *in vivo.* In particular, S protein in GBS appears to be required for proper anchoring of the surface polysaccharide capsule, a classical virulence determinant among GBS strains. Alterations within the GBS surface-anchored virulome extend beyond capsular retention to protein and glycoprotein-based surface characteristics and extracellular proteome of GBS important for virulence. A GBS S protein null strain was highly impaired in its ability to resist neutrophil and whole blood clearance and to cause invasive disease *in vivo.* Proteome analysis of the organs revealed that the S protein deletion mutant elicited a more robust systemic host response compared to the WT strain, suggesting that rapid clearance of the bacteria is associated with efficient recognition of the bacteria resulting from the absence of S protein. Further analysis of brain tissues showed increased clathrin-related proteins during WT GBS infection.

In deleting the S protein homolog gene locus (*ess*) we identified altered surface chemistry and aggregation phenotypes, which were restored when the mutant was complemented using a vector with S protein expressed *in trans*. Altered surface chemistry presented as increased hydrophobicity, which we linked to reduced capsular retention. This linkage was further solidified by the downregulation of capsular biosynthesis protein in extracellular proteomic analysis. Furthermore, S protein deletion led to intriguing changes in GBS pigment with retained hemolytic capabilities, which are phenotypes commonly regarded as interdependent. Our results also demonstrate that S protein occupies a central role in surface homeostasis of GBS. Given the pleiotropic impact of S protein deletion of GBS physiology, the impaired *in vivo* virulence phenotype identified is likely reflective of the cumulative surface changes to the bacteria. S protein deletion sensitized bacteria to a robust immune response in the spleen including an increase in the abundance of proteasome components, an important contributor to antigen presentation during the development of adaptive immunity. While the S protein deletion strain is efficiently cleared from the system, the bacteria may nevertheless be processed to promote long-term adaptive immunity against GBS (Lemire et al., 2012; Uchiyama et al., 2019; Zhu et al., 2021).

Additional studies are needed to more completely understand the role of the S protein homolog in GBS pathophysiology and how alterations in the GBS surface resulted in increased vulnerability to detection and killing by host immune modalities. Future work to probe the relationship of S protein to the full catalog of known GBS virulence determinants will help determine its viability as an anti-virulence or vaccine target.

## Data Availability Statement

The datasets presented in this study can be found in online repositories. The names of the repository/repositories and accession number(s) can be found in the article/Supplementary Material.

## Ethics Statement

The animal study was reviewed and approved by the Animal studies were conducted under protocols approved by the UC San Diego Institutional Animal Care and Use Committee (IACUC). Written informed consent was obtained from the individual(s) for the publication of any potentially identifiable images or data included in this article.

## Author Contributions

AC, VN, and DG conceived of the study. AC, SU, VN, and DG designed the experiments. AC, SU, CSan, and CSau executed the experiments. AC and SU performed the formal data analysis. AC wrote the manuscript. AC, SU, CSan, CSau, VN, and DG edited the manuscript. DG and VN secured funding for this study. All authors contributed to the article and approved the submitted version.

## Conflict of Interest

The authors declare that the research was conducted in the absence of any commercial or financial relationships that could be construed as a potential conflict of interest.

## Publisher’s Note

All claims expressed in this article are solely those of the authors and do not necessarily represent those of their affiliated organizations, or those of the publisher, the editors and the reviewers. Any product that may be evaluated in this article, or claim that may be made by its manufacturer, is not guaranteed or endorsed by the publisher.

## Footnotes

1
https://software.broadinstitute.org/morpheus
