# Supplementary figures and images for: The S Protein of Group B Streptococcus Is a Critical Virulence Determinant That Impacts the Cell Surface Virulome
Source: Front Microbiol. 2021 Oct 14;12:729308. doi: 10.3389/fmicb.2021.729308 (PMC8551713; doi:10.3389/fmicb.2021.729308)

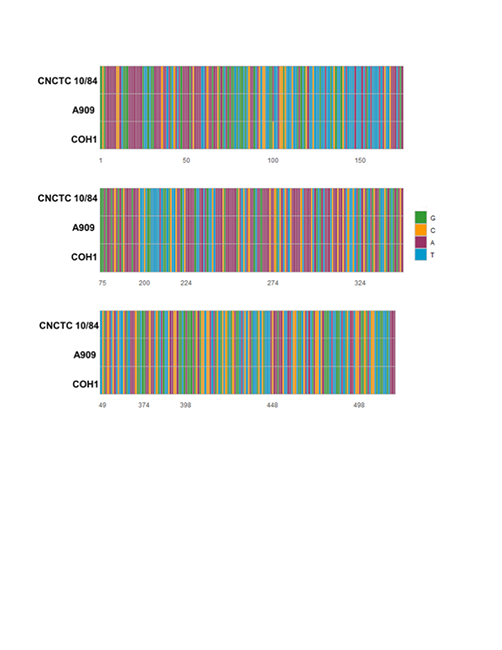

Supplement: Supplementary file 1 [file Image_1.tiff]

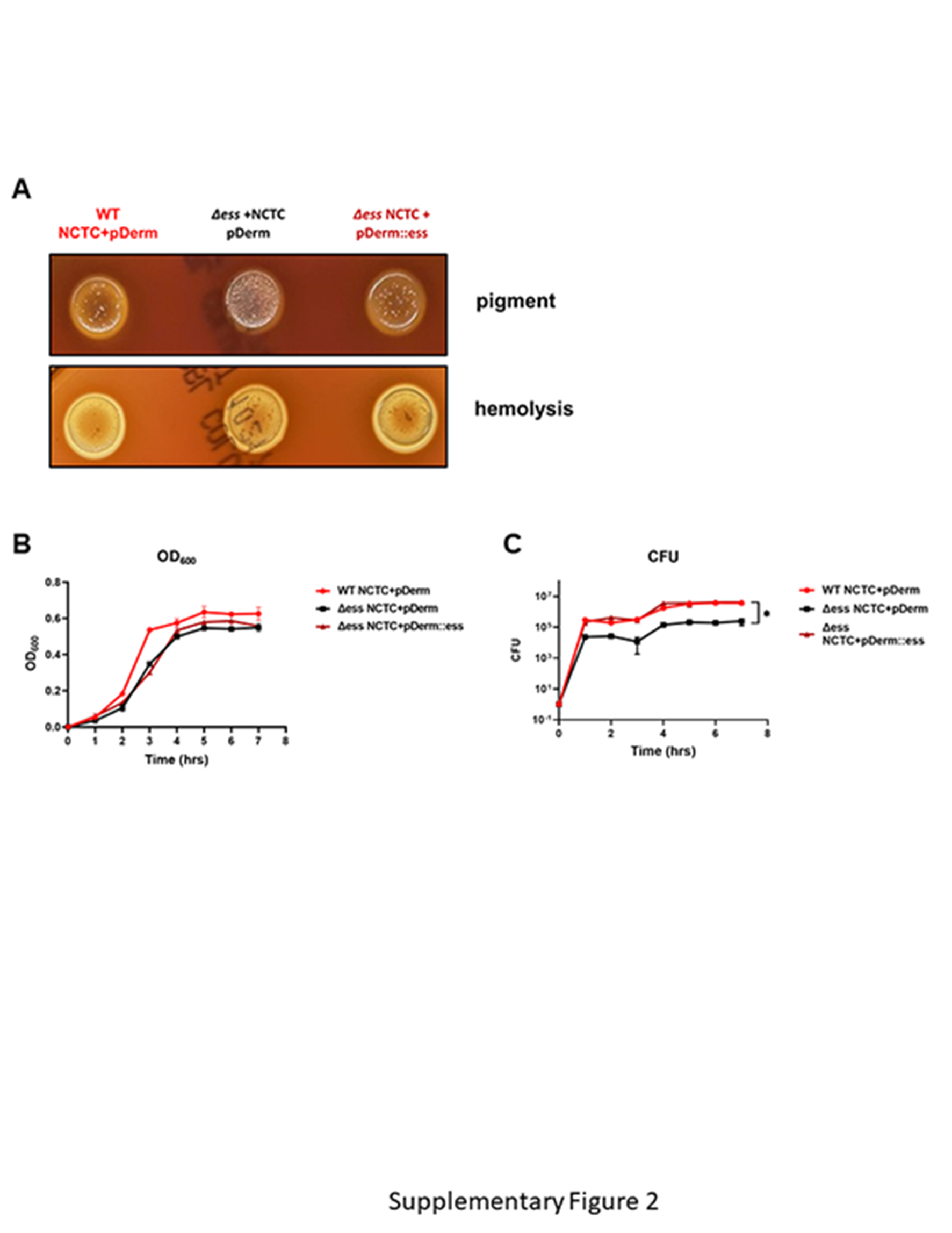

Supplement: Supplementary file 2 [file Image_2.TIF]

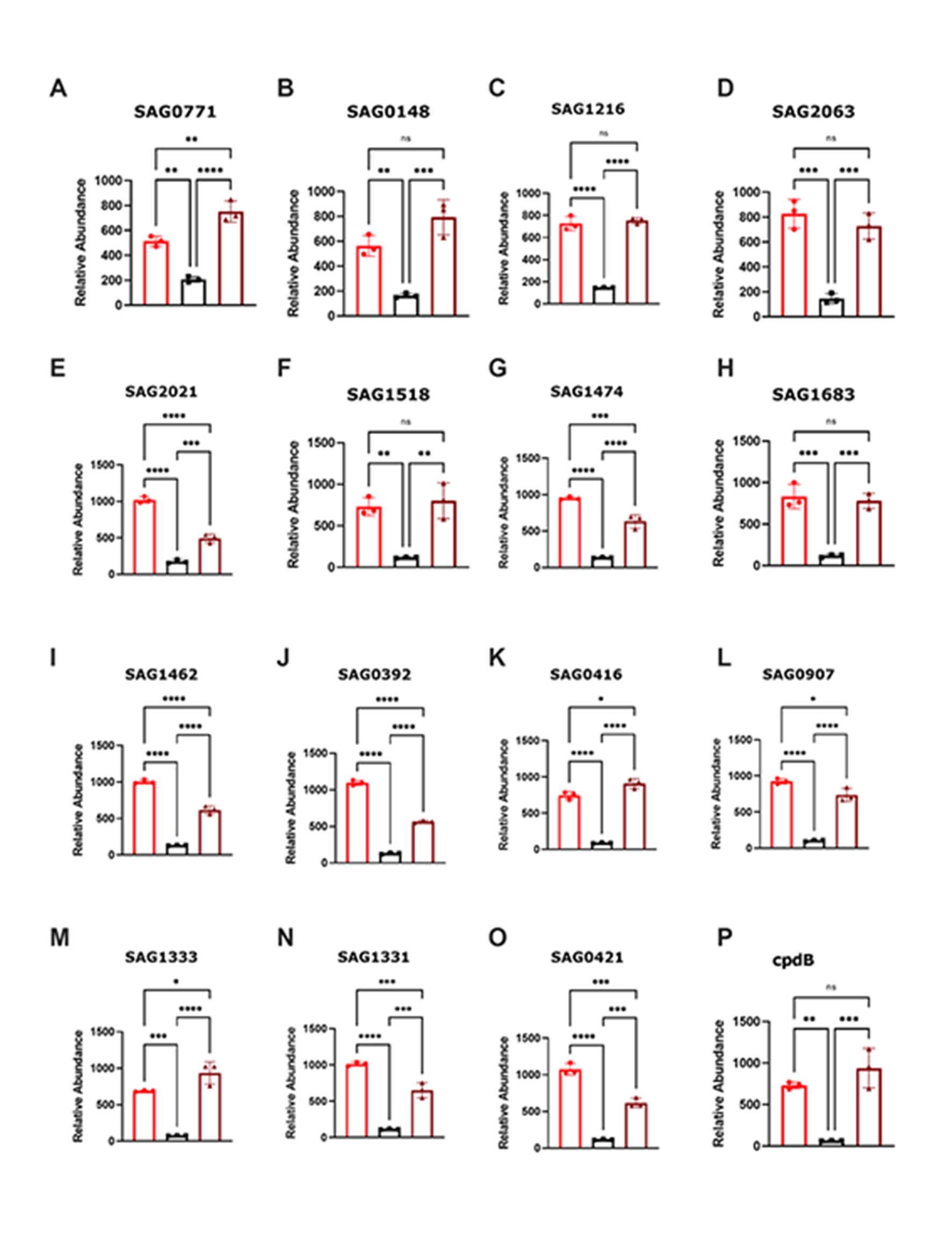

Supplement: Supplementary file 3 [file Image_3.TIF]

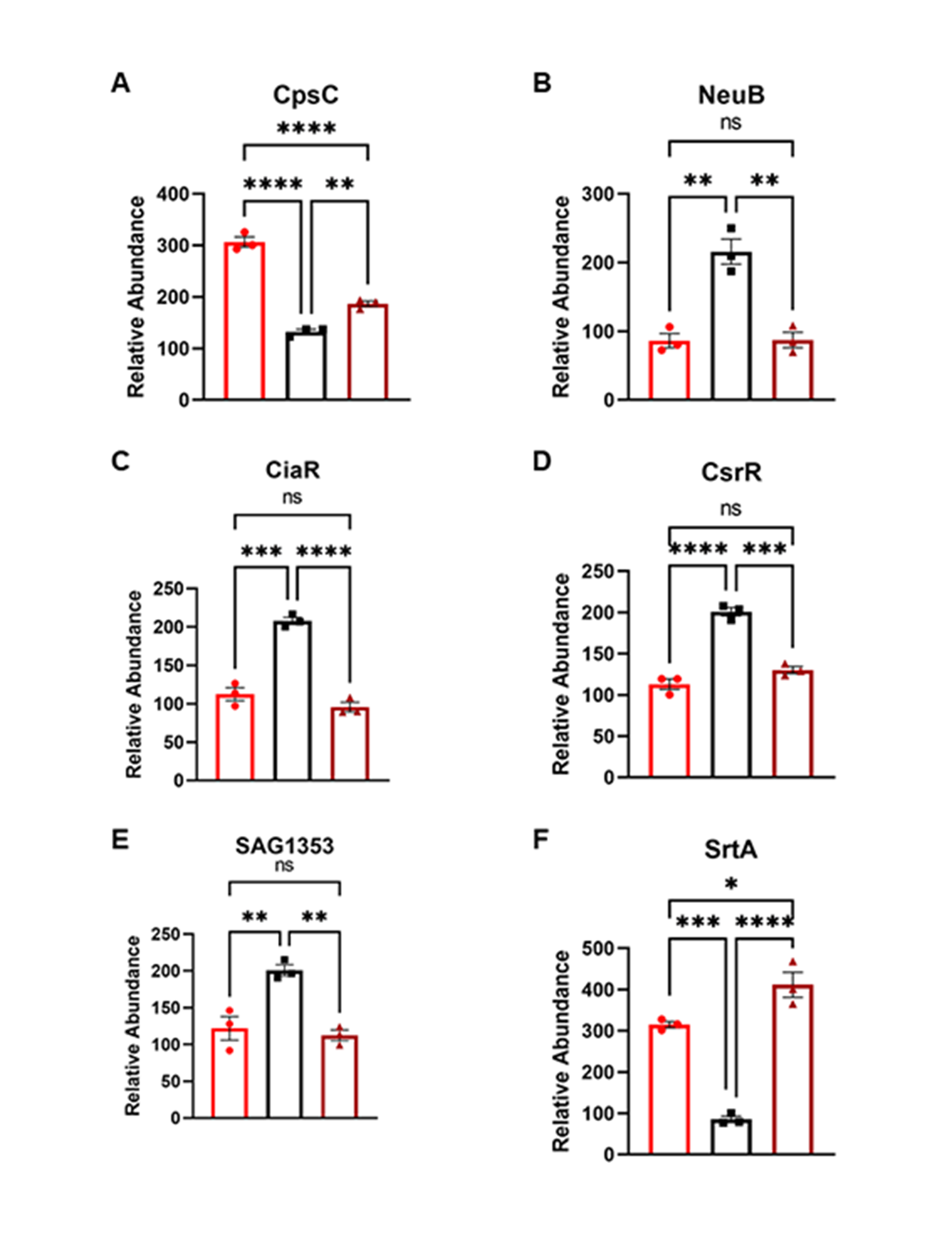

Supplement: Supplementary file 4 [file Image_4.TIF]

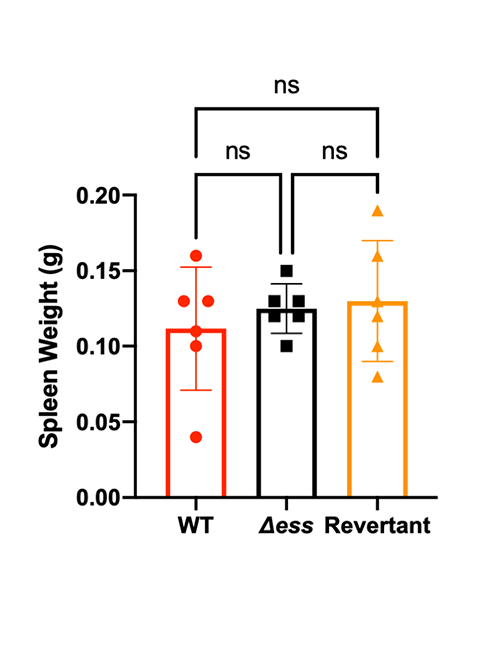

Supplement: Supplementary file 5 [file Image_5.tiff]
