# Supplementary material for: The S Protein of Group B Streptococcus Is a Critical Virulence Determinant That Impacts the Cell Surface Virulome
Source: Front Microbiol. 2021 Oct 14;12:729308. doi: 10.3389/fmicb.2021.729308 (PMC8551713; doi:10.3389/fmicb.2021.729308)
Supplement: Supplementary file 8 [file Data_Sheet_3.docx]

**Supplemental Figure Legends**

Supplemental Figure1: Multiple sequence alignment of GBS S protein sequences

A). Multiple sequence alignment of GBS S protein (*ess*) demonstrating degree of conservancy in GBS strains represented by CNCTC 10/84, A909, and COH1 strains.

Supplemental Figure 2: GBS strain hemolysis and pigment

A). Photo demonstrating pigment and hemolysis of WT, S protein deleted, and complemented strains cultured on blood agar.

B). Optical density growth curve analysis of WT, S protein deleted, and complemental strains. OD_600_ was measure at 1-hour timepoints.

C). CFU enumeration growth curve analysis of WT, S protein deleted, and complemented strains collected at 1 hour timepoints. Significance was determined using one-way ANOVA with Tukey’s multiple comparison test (* p-value<0.05; ** p-value<0.01; *** p-value<0.001; **** p-value<0.0001;ns non-significant).

Supplemental Figure 3: Cell wall-associated protein abundance for proteins significantly decreased in comparison of Δ*ess* to either WT or complemented strains.

A-P). Relative abundance for pSORTb-designated cell wall proteins significantly decreased in comparisons of Δ*ess* to either WT or complemented strain surfomes. Significance was determined using one-way ANOVA with Tukey’s multiple comparison test (* p-value<0.05; ** p-value<0.01; *** p-value<0.001; **** p-value<0.0001;ns non-significant).

Supplemental Figure 4: Protein abundances for proteins differentially abundant in culture supernatant of WT, S protein deleted, and complemented strains.

A-F). Relative abundance of named proteins in culture supernatant of WT, S protein deleted, and complemented strains. Significance was determined using one-way ANOVA with Tukey’s multiple comparison test (* p-value<0.05; ** p-value<0.01; *** p-value<0.001; **** p-value<0.0001;ns non-significant).

Supplemental Figure 5: Spleen weight for infected animals

A). Spleen weight for animals infected with WT, Δ*ess,* or revertant strains of GBS. Significance was determined using one-way ANOVA with Tukey’s multiple comparison test (ns non-significant).
